# 20 years of porous tantalum in primary and revision hip arthroplasty—time for a critical appraisal

**DOI:** 10.1080/17453674.2018.1463007

**Published:** 2018-05-04

**Authors:** Nils Hailer

**Affiliations:** Orthopaedics/Department of Surgical Sciences Uppsala University Hospital

After almost 2 decades of clinical use, porous tantalum has become an important component in the orthopedic surgeon’s toolbox, and its most common use today is as acetabular cups, cages, or augments in revision arthroplasty. For many revision surgeons, porous tantalum has significantly changed their practice when addressing more complex acetabular bone loss. However, now porous tantalum is also increasingly used as a coating for cups used in primary total hip arthroplasty (THA). It is time for a critical appraisal of porous tantalum in hip arthroplasty, and this issue of Acta Orthopaedica contains an article that investigates the use of porous tantalum cups in primary THA (Laaksonen et al. 2018).

## Porous tantalum in revision THA

Tantalum was first described in 1802 by the Swedish chemist Anders Ekeberg, who isolated it from the rare mineral tantalite. In modern times, pure tantalum has been manufactured into 3-dimensional, macroporous structures that closely resemble cancellous bone, so-called “trabecular metal” (TM). Its mechanical and biological properties make porous tantalum well suited for orthopedic applications:

- Mechanically, a fairly unique combination of high elasticity and a high coefficient of friction (Levine et al. 2006) gives porous tantalum implants their much appreciated “grip” in situations where primary stability is critical but difficult to achieve, such as in acetabular revision surgery with a high degree of bone loss and sclerotic acetabular rims.
- Biologically, tantalum and titanium share a very pronounced thrombogenic potential, a property that may be important since blood clot formation is one of the first and essential steps in bone healing, and this distinguishes tantalum and titanium from other metals such as nickel (Hong et al. 2005). Furthermore, porous tantalum in the shape of “trabecular metal” has a very high porosity of 75–85%, and its pore size of more than 500 µm is also relatively large (Balla et al. 2010). These properties may at least in part explain the osteoconductivity that is observed in different in vivo models investigating the integration of porous tantalum into host bone (Levine et al. 2006).

The first clinical reports on porous tantalum shells were on patients with technically demanding acetabular defects (Sporer and Paprosky 2006, Flecher et al. 2008). Further applications included the combination of porous tantalum shells with differently shaped augments or cages that are manufactured from the same material, again for use in large acetabular defects or even in pelvic discontinuity (Abolghasemian et al. 2014). Comparative retrospective studies indicate that porous tantalum cups used in revision situations are at least as good as conventional uncemented titanium cups (Jafari et al. 2010, Mohaddes et al. 2015), and that they can be superior to Müller acetabular reinforcement rings in terms of a reduced risk of aseptic loosening (Brüggemann et al. 2017).

On the other hand, dislocation seems to be a recurrent issue after the use of porous tantalum shells in revision THA (Skytta et al. 2011, Brüggemann et al. 2017), a problem that has not been investigated in depth. This instability could be related to difficulties in restoring a correct center of rotation‚ or in less than optimal abduction and anteversion angles, possibly related to the strategy of “going for bone” as opposed to reconstructing the acetabular bed, as in the old-fashioned techniques. The combination of dual-mobility cups with porous tantalum shells may reduce the problem of instability (Brüggemann et al. 2018), but this hypothesis needs further investigation.

## Porous tantalum cups in primary THA

The next step of using porous tantalum as a cup material even in primary arthroplasty surgery was both logical and tempting, and initial medium-term reports showed good results (Malizos et al. 2008, Macheras et al. 2009). Subsequently, 2 randomized controlled trials compared porous tantalum with hemispherical titanium cups in primary THA. When measured by radiostereometry, porous tantalum cups had slightly better initial stability than titanium fiber-mesh cups, but they were accompanied by a similar degree of periprosthetic bone loss (Baad-Hansen et al. 2011). A randomized controlled trial comparing porous tantalum monoblock with porous-coated titanium monoblock cups found fewer radiolucencies around the porous tantalum cups (Wegrzyn et al. 2015), but of course none of these trials was designed or powered to detect differences in the risk of revision.

Large-scale registry studies provide the opportunity to investigate survival data derived from large cohorts, and—unexpectedly—both the Australian (Australian Orthopaedic Association National Joint Replacement Registry (AOANJRR) 2017) and the Swedish arthroplasty registries (Swedish Hip Arthroplasty Register 2016) repeatedly reported a higher-than-expected revision risk for porous tantalum cups that were used in primary THA, in part due to an increased risk of dislocation. In contrast, a recently published registry study from the England and Wales National Joint Registry that compares porous tantalum-coated cups with titanium-coated cups from the same manufacturer finds a lower risk of revision due to aseptic loosening in the group of patients operated with porous tantalum cup (Matharu et al. 2018). Authors from the Australian and Swedish joint registries have now collaborated in a joint effort to compare the outcome after the use of porous tantalum cups in primary THA with that of other common uncemented cups. In the present issue of Acta Orthopaedica, Laaksonen et al. (2018) report a 1.5-fold higher risk of revision for the porous tantalum-coated cups.

## Porous tantalum—what needs to be done?

Porous tantalum cups undoubtedly confer highly desirable effects in terms of excellent initial and long-term stability in demanding acetabular revision surgery, and they have certainly come to stay. Further studies are needed to investigate whether a combination of porous tantalum shells with dual-mobility systems reduces instability after complex revision procedures.

However, what is good in revision surgery is not necessarily superior in standard primary THA surgery. Could it be that—in the context of primary THA—the terrific grip of porous tantalum cups makes them more likely to jam in suboptimal positions, maybe retroverted, or steeper than intended, but that the surgeon refrains from correcting their position simply because they are difficult to get out? This is entirely speculative, and a systematic analysis of the mechanisms underlying the potentially increased risk of dislocation after the use of porous tantalum cups has not yet been done.

The orthopedic community will have to keep an eye on the evolving evidence in order to critically assess whether we should continue to be as tantalized by tantalum in primary THA as we are in complex revision situations.
